# Experimental ‘Jet Lag’ Inhibits Adult Neurogenesis and Produces Long-Term Cognitive Deficits in Female Hamsters

**DOI:** 10.1371/journal.pone.0015267

**Published:** 2010-12-01

**Authors:** Erin M. Gibson, Connie Wang, Stephanie Tjho, Neera Khattar, Lance J. Kriegsfeld

**Affiliations:** 1 Department of Psychology, University of California, Berkeley, California, United States of America; 2 Helen Wills Neuroscience Institute, University of California, Berkeley, California, United States of America; Vanderbilt University, United States of America

## Abstract

**Background:**

Circadian disruptions through frequent transmeridian travel, rotating shift work, and poor sleep hygiene are associated with an array of physical and mental health maladies, including marked deficits in human cognitive function. Despite anecdotal and correlational reports suggesting a negative impact of circadian disruptions on brain function, this possibility has not been experimentally examined.

**Methodology/Principal Findings:**

In the present study, we investigated whether experimental ‘jet lag’ (i.e., phase advances of the light∶dark cycle) negatively impacts learning and memory and whether any deficits observed are associated with reductions in hippocampal cell proliferation and neurogenesis. Because insults to circadian timing alter circulating glucocorticoid and sex steroid concentrations, both of which influence neurogenesis and learning/memory, we assessed the contribution of these endocrine factors to any observed alterations. Circadian disruption resulted in pronounced deficits in learning and memory paralleled by marked reductions in hippocampal cell proliferation and neurogenesis. Significantly, deficits in hippocampal-dependent learning and memory were not only seen during the period of the circadian disruption, but also persisted well after the cessation of jet lag, suggesting long-lasting negative consequences on brain function.

**Conclusions/Significance:**

Together, these findings support the view that circadian disruptions suppress hippocampal neurogenesis via a glucocorticoid-independent mechanism, imposing pronounced and persistent impairments on learning and memory.

## Introduction

Frequent transmeridian travel, rotating shift work schedules, and irregular sleep patterns result in an incongruence between the endogenous circadian timing system and the external environment [1], [2], [3], [4]. This loss of synchrony is associated with a number of clinical pathologies, including a higher incidence of cancer [5], [6], diabetes [7], hypertension and cardiovascular disease [8], [9], reduced fertility and fecundity [10], [11], and an exacerbation in a number of pre-existing psychological pathologies [12], [13] relative to individuals with consistent schedules. Most relevant to the present series of studies, several lines of investigation using human and animal models suggest a pronounced influence of circadian timekeeping on learning and memory [14], [15], [16], [17].

In mammals, the master circadian pacemaker is located in the suprachiasmatic nucleus (SCN) in the anterior hypothalamus [18], [19]. The SCN generates endogenous oscillations with a period of approximately, but not precisely, 24 hours, resulting in a desynchrony between internal and environmental time in the absence of an external synchronizing cue. This desynchrony is prevented through entrainment, with light being the primary zeitgeber (time giver; ZT) in mammals [20]. At the cellular level, circadian rhythms are generated by 24-hour autoregulatory transcriptional/translational feedback loops consisting of ‘clock’ genes and their protein products [21], [22], [23], [24]. Importantly, clock gene expression is ubiquitous and allows the CNS and periphery to exhibit system-specific rhythms in daily activity, a necessity for optimal health and functioning.

Several correlational studies suggest an association between circadian disruptions and impaired cognitive function in humans [2]. For example, learning and memory deficits and reduced temporal lobe volume are observed in chronically jet-lagged female flight attendants relative to controls [16], [17]. These cognitive deficits are associated with elevated circulating cortisol concentrations relative to flight attendants permitted recovery following transmeridian travel [17]. However, in order to establish a cause-effect relationship between alterations in circadian timing and learning and memory deficits, experimental studies in which circadian perturbations are controlled and applied to a homogenous population are required.

In addition to the effects of circadian timing, numerous lines of evidence point to a strong association between neurogenesis and learning and memory, suggesting that new cell birth/maturation might be negatively affected by disruption of daily rhythms. For example, newly born hippocampal cells markedly increase following a hippocampus-dependent learning task [25], [26], [27]. Importantly, learning tasks that are hippocampus-*independent* do not result in increased dentate gyrus neurogenesis [25], [28]. More recent studies using antimitoic and DNA alkylating agents, irradiation, targeted viral vector, and genetic approaches to more specifically disrupt neurogenesis provide further support for the importance of new neuron proliferation/maturation in learning and memory [29], [30], [31], [32].

Given the impact of circadian perturbations on the stress and reproductive axes [10], [11], and established effects of glucocorticoids and estrogen on hippocampal cell proliferation/neurogenesis [33], [34] and learning and memory [34], [35], [36], [37], [38], [39], it is possible that disruptions in circadian timing negatively impact cognitive function through glucocorticoid- and/or ovarian hormone-dependent changes in neurogenesis. Alternatively, circadian disruption may impact brain function more directly, as mice lacking one of the core clock genes, *Period2*, that drives circadian rhythms at the cellular level, exhibit alterations in neural/progenitor cell proliferation in the hippocampus [40]. This finding suggests that the state of the circadian system may directly affect the cell cycle and cell proliferation [41].

In the present series of studies, we sought to establish whether or not disruptions in circadian timing impact learning and memory. Additionally, given the association between adult neurogenesis and learning and memory, we examined the possibility that hippocampal cell proliferation and neurogenesis are affected by disturbances in circadian timing. We examined dentate gyrus cell proliferation and neurogenesis in female Syrian hamsters exposed to 4 weeks of twice weekly phase advances in the LD cycle (i.e., a 6-hr experimental ‘jet lag’). Similar manipulations have been previously used to assess the impact of experimental jet lag on mortality [42] and tumor progression [43]. We intentionally chose repeated phase advances for this initial characterization because these manipulations require significantly more time for behavioral and physiological re-entrainment than phase delays [3], [42], [44], [45], [46]. Likewise, these behavioral manipulations allow for the study of circadian disruption on variables of interest without invasive surgical manipulations or global disruption of the molecular circadian clockwork. The relative contribution of alterations in the stress and reproductive axes to any observed deficits were controlled through adrenalectomy or ovariectomy and hormone replacement (corticosterone or estrogen, respectively). Hippocampal-dependent memory was assessed using a conditioned place preference (CPP) paradigm during the time of circadian perturbations, and well after the cessation of jet lag, to explore whether or not any impact on cognitive functioning persists following re-entrainment.

## Materials and Methods

### Ethics Statement

All animal experiments were in accordance with NIH guidelines regarding the care and use of animals and all protocols were approved by the Institutional Animal Care and Use Committee of the University of California, Berkeley (Protocol R295).

### Animals

Adult (>60 days of age) female LVG hamsters (*Mesocricetus auratus*; Charles River, Wilmington, MA) were maintained on a 14∶10 light∶dark (LD) cycle (lights on at 0700 h) prior to the onset of all experiments, with a light intensity ranging from 100–300 lux at the level of each cage. All animals were maintained in a colony room at 23±1°C and provided with *ad libitum* access to water and food. Estrous cyclicity was monitored for all animals by daily inspection for preovulatory vaginal discharge [47]. Only animals with regular, 4-day estrous cycles were used in the experiments. The first cohort of hamsters either remained intact or was ovariectomized or adrenalectomized to assess the influence of estrogen and glucocorticoids on cell proliferation and neurogenesis (n = 27). All surgeries were conducted under isoflurane anesthesia. Ovariectomized hamsters received a SILASTIC brand capsule (Downing Corning Corp., Midland, MI; 10 mm length, 1.45 inner diameter, 1.93 out diameter) containing powdered 17-β estradiol (OVX+E_2_). These capsules result in proestrous concentrations of plasma estradiol [48]. Adrenalectomized hamsters were given a solution of 0.9% saline, 5% sucrose and corticosterone (25 µg per ml of 0.9% saline; Sigma) to mimic basal glucocorticoid concentrations and maintain electrolytes (ADX). This treatment results in basal levels of corticosterone, the dominant hamster glucocorticoid in non-stressed animals [48], [49], [50]. Two weeks after surgery, hamsters were placed into their respective lighting conditions. A second cohort was used to investigate the impact of jet lag on learning and memory (n = 20). The final cohort of hamsters was used to assess the impact of jet lag on behavior and the stress axis (n = 14).

### Jet Lag and Hippocampal Cell Proliferation/Neurogenesis

Hamsters either remained intact or were adrenalectomized and provided with basal corticosterone concentrations or ovariectomized and provided with proestrous estradiol concentrations. Half (n = 4–5/group) of the animals from each condition were exposed to a 6-hr phase advance every 3 days for 25 days (Jet Lag) while the other half remained in a 14∶10 LD (lights on at 0700 hr) cycle for the same duration as jet-lagged animals (Control). All animals were injected with the thymidine analog, bromodeoxyuridine (BrdU), to label the dividing cell population. BrdU (50 mg/kg body weight; Sigma) was injected intraperitoneally (i.p.) 7 hours after lights on, one day after every second phase advance (i.e., every 6 days) for the jet lag condition or at the same time and day for control hamsters (**Figures S1 and S2**). Multiple injections of BrdU were used to estimate the total population of newly-generated cells throughout the 25-day temporal disruption, as well as to maximize the number of cells surviving until differentiation [51], [52].

Hamsters were then anesthetized with sodium pentobarbital (200 mg/kg) and perfused transcardially with 150 ml of 0.9% saline followed by 300 ml of 4% paraformaldehyde in 0.1 M PBS (pH 7.4) 24 hours after the last BrdU injection. Brains were postfixed in 4% paraformaldehyde for 3 hours at 4°C and cryoprotected in 30% sucrose in 0.1 M PBS overnight (**Figure S2**).

### Histological Procedures, Microscopy, and Quantification

Brains were sectioned in the coronal plane at 40 µm thickness using a cryostat (Leica, CM3050-S, Leica Microsystems Inc., Bannockburn, IL). For BrdU immunofluorescence, sections were rinsed in 0.4% Triton X-100 (PBT) followed by 10 min in 0.9% saline. Sections were then denatured in 2 M HCl for 30 min at 37°C, rinsed in PBT, and incubated in normal donkey serum (1∶50; Jackson ImmunoResearch) in PBT for 1 hr. Sections were then co-incubated for 48 hr at 4°C in rat anti-BrdU (1∶1000; Accurate Chemical), guinea pig anti-glial fibrillary acidic protein (GFAP) (1∶1000; Advanced Immunochemical), and mouse anti-neuronal nuclei protein (NeuN) (1∶1000; Chemicon). Following incubation in the primary antibodies, sections were rinsed in PBT and incubated in the dark for 1 hr with DAPI which binds strongly to DNA and labels cellular nuclei (1∶1000: Sigma), CY2 donkey anti-rat (1∶500; Jackson ImmunoResearch), CY5 donkey anti-guinea pig (1∶500; Jackson ImmunoResearch), and CY3 donkey anti-mouse (1∶500; Jackson ImmunoResearch) to visualize BrdU, GFAP, and NeuN, respectively.

GFAP was used to assess gliogenesis while NeuN, a vertebrate nervous system nuclear protein ubiquitous in the CNS, was used to label mature neurons. This same protocol was followed for proliferating cell nuclear antigen (PCNA), a co-factor for DNA polymerase and a convenient endogenous marker for newly proliferated cells, immunofluorescence using mouse anti-PCNA (1∶4000; Santa Cruz) as the primary antibody. PCNA was visualized with CY3 donkey anti-mouse (1∶500; Jackson ImmunoResearch). Sections were then rinsed, mounted on gelatin-coated slides, dehydrated with a graded series of alcohols, and coverslips were applied.

All cell counting was performed by individuals blind to the experimental conditions. All sections were counted using a Zeiss Z1 microscope (Carl Zeiss, Thornwood, NY) at 400× using the standard wavelengths for FITC (485 nm), CY3 (546 nm), CY5 (640 nm), and DAPI (359 nm). For BrdU-positive cells or PCNA-positive cells, every 12^th^ unilateral section throughout the extent of the dentate gyrus (including the subgranule zone, the granule zone and the hilus) was counted, excluding those cells in the outermost field of focus. Volume reconstruction was conducted by multiplying the number of BrdU-positive or PCNA-positive cells per dentate gyrus by 24 to estimate the total number of labeled cells per brain [50]. The volume of the analyzed region was determined using Cavalieri's principle with NIH ImageJ software [53]. 25 BrdU-labeled cells from at least 4 sections/animal were randomly chosen and assessed for double-labeling with NeuN or GFAP [54]. Images were digitally captured at 400× in 8-bit greyscale using a cooled CCD camera (Zeiss). At least 20% of those BrdU-labeled cells assessed for double-labeling were analyzed in confocal scans to ensure that counts using conventional microscopy did not result in false positives. In all cases, those cells identified as double-labeled at the conventional microscopy level were identified as double-labeled in the confocal microscopy analysis.

### Assessment of Learning and Memory: Conditioned Place Preference

Intact, adult, female LVG hamsters (4–5 weeks of age) were used to assess hippocampal-dependent learning and memory using the conditioned place preference (CPP) paradigm (n = 20), considered an ideal hippocampal-dependent memory test in Syrian hamsters. Hamsters do not perform well on other established hippocampal-dependent memory tests, including the Morris water maze or the Olton radial arm maze [15], [55]. In the CPP paradigm animals learn an association between a specific context and a rewarding stimulus (a running wheel in the present case) [15], [56]. Learning is indicated by an increase in dwell time - the total time spent in the context previously paired with the rewarding stimulus compared to the non-rewarded. The animal was considered to enter a chamber when both forepaws were within the chamber. Estrous cyclicity was monitored daily for 2 wks prior to testing to ensure that pre-testing occurred on the same day of the estrous cycle for all hamsters to control for estrogenic effects on learning and memory [57]. Stainless steel wheels, 17.5 cm diameter, were placed in the home cages of all hamsters 2 wks prior to onset of the testing to acclimate the animals to wheel running. All testing occurred in a dark room illuminated by dim red light to encourage exploration, particularly of the white compartment.

The CPP apparatus included two boxes (60×45×40 cm), one white and one black, connected by a clear pathway (30×25×18 cm). Sliding partitions that matched the color of the compartments were used to isolate animals in one of the chambers during training. To further distinguish the boxes, a unique odor was placed into a wall-mounted plastic container matching the color of each chamber. Before each session a cotton ball saturated with either 0.5% isoamyl acetate or eucalyptus oil was added to each of the chambers. Each box was associated with one of the odors for the duration of the experiment. Between tests the compartments were cleaned with 70% ETOH. Tests for context preferences were determined by recording the total amount of dwell time in each context. The CPP consisted of three phases: Pretest, Training and Probe trials.

Pretests commenced during estrus with Probe trials occurring on diestrus. Hamsters were exposed to the CPP apparatus at the same time of day for the entire behavioral protocol, with all Pretest, Training, and Probe trials occurring within 4 hrs of lights off. Because Syrian hamsters show a place preference for a reward-paired context only when the training and probe trials occur at the same time of day (ZT) or the same circadian time (CT) [15], we elected to train hamsters at the same ZT (i.e., within 4 hrs of lights off). Pretest 1 occurred on Days 13 through 16 of the jet lag paradigm, depending on the estrous state of the animals. Probe 1 occurred on Days 21 through 25 (**Figure S2**). After Probe 1, jet-lagged animals were returned to a static light∶dark cycle (14∶10 LD). Pretest 2 was conducted after *all* hamsters were maintained in a *static* LD cycle for 28 days to determine if jet lag had lingering effects on learning and memory long after cessation of the temporal disruption. Control animals were housed in 14∶10 LD for the duration of the experiment. Specific procedural details are below:

#### CPP test 1

Pretest 1 - Animals were placed into the clear center partition and allowed to explore the entire apparatus for 10 min. Videos were scored to determine the total amount of time spent in each compartment. If the hamster exhibited a preference for one of the boxes (white or black), the wheel was assigned to the box opposite their preference to remove the possibility of bias for a particular box for each individual animal. Video recordings were also examined to calculate the total amount of time the animals were active or ambulating to ensure that control and jet-lagged hamsters were equally motivated to explore the apparatus. Initial Training – Hamsters were trained for 25 min/day with the wheel placed in the compartment assigned to each animal based on Pretest preferences. Each animal received 4 training sessions in which it was confined to the box containing the wheel and 4 training sessions in which it was confined to the box without the wheel (alternating days). Probe 1 – On the test day, the wheel was not present in the apparatus. Hamsters were tested to determine if they retained a memory for the chamber paired with the wheel by placing the hamsters into the clear center partition and permitting them to freely explore the entire apparatus for 10 min. Hamsters were videotaped and the total amount of time spent in either the black or white compartment was recorded as in Pretest 1.

#### CPP test 2

Pretest 2 – One month after all hamsters were placed into a static LD cycle, they were tested to determine if they maintained a memory for the previous learning task. Hamsters were placed into the center partition and permitted to freely explore the apparatus for 10 min. During this test, no wheel was present to assess whether animals recalled the location of the wheel during CPP Test 1. Videos were assessed and the amount of time spent in each chamber was recorded as in Pretest 1. Reversal Training – For this training experience, the wheel was now placed into the opposite chamber from that which each individual hamster experienced during the first training session. All other training conditions were implemented as in the Initial CPP Test 1 Training Session. Reversal Probe – To determine if hamsters learned the new location of the wheel, a probe trial was conducted in which the wheel was removed. As in Probe 1, hamsters were released into the apparatus and allowed to freely explore for 10 min. Video recordings were analyzed and the total amount of time spent in each compartment was recorded as in CPP Test 1, Probe 1.

### Jet Lag Treatment, Behavioral Monitoring, and Hypothalamo-Pituitary Adrenal (HPA) Axis Activation

Hamsters were either exposed to the jet lag condition (n = 7) or to a fixed LD cycle (n = 7). Locomotor behavior was monitored for all animals using an infrared monitoring system (Data Sciences; St. Paul, MN) mounted to the wire lids on each cage. All movement in the cage was detected by interruptions in the infrared beam and relayed to a computer. Cumulative counts were recorded every 10 min and analyzed using Dataquest 3 software (Data Sciences; St. Paul, MN). The power of all rhythms was assessed using Fourier analysis (Clocklab) in which an animal was considered rhythmic when its highest peak occurred approximately 1 cycle/day. Clocklab software was also used to determine the nocturnality index and *alpha* for the jet-lagged animals prior to the onset of the phase advancements (fixed LD), as well as during the jet lag paradigm on days 2–4, 15–17, and 21–23. The nocturnality index is the ratio of the time active during the dark phase compared to the total time active over 24 hrs. Animals that are more active during the dark phase will have a higher nocturnality index. *Alpha* is defined as the difference between activity onset and activity offset. Activity onset was defined as the first bout of sustained activity after a period of 2 hrs with less than 20 min of activity. Activity offset was defined as the final bout of activity before a period of 2 hrs with less than 20 min of activity. In addition to monitoring the activity rhythms of the hamsters, cortisol concentrations were assessed throughout the 25-day jet lag schedule from blood samples collected through the retroorbital sinus. Animals were anesthetized using isoflurane, and blood samples were collected 7 hrs into the rest phase based on their activity profile for all hamsters on days 2, 8, 15, and 25 of the jet lag paradigm (**Figure S2**). Cortisol was measured in 25 µl aliquots of serum using RIA kits from ICN Biomedicals, Inc., Diagnostic Division (Costa Mesa, CA). The cortisol assay has been validated previously for use in Siberian hamsters [58]. The intraassay coefficient of variation for cortisol was 1.39%, and the interassay coefficient of variance was 12.5%. The minimum detectable cortisol concentration was 0.25 µg/dl [59].

### Statistical Analyses

Group mean differences in cell counts were analyzed using analyses of variance (ANOVA) with Tukey or Tukey-Kramer post hoc tests to examine pairwise differences. Cortisol data were analyzed using a two-way repeated measures ANOVA while activity data were analyzed using a one-way repeated measures ANOVA. Student's *t*-tests were performed to assess chamber preference, total duration of time active, and learning and memory for the paired context in the Conditioned Place Paradigm. A Levene test for homogeneity of variance was performed to assess differences in variability between groups during Probe 1. A Pearson Product Moment Correlation was used to assess the correlation between amount of light exposure prior to blood sampling and cortisol concentrations. Findings were considered significant when *P*<0.05.

## Results

### Jet Lag Decreases Hippocampal Cell Proliferation and Neurogenesis

**Figure 1:**
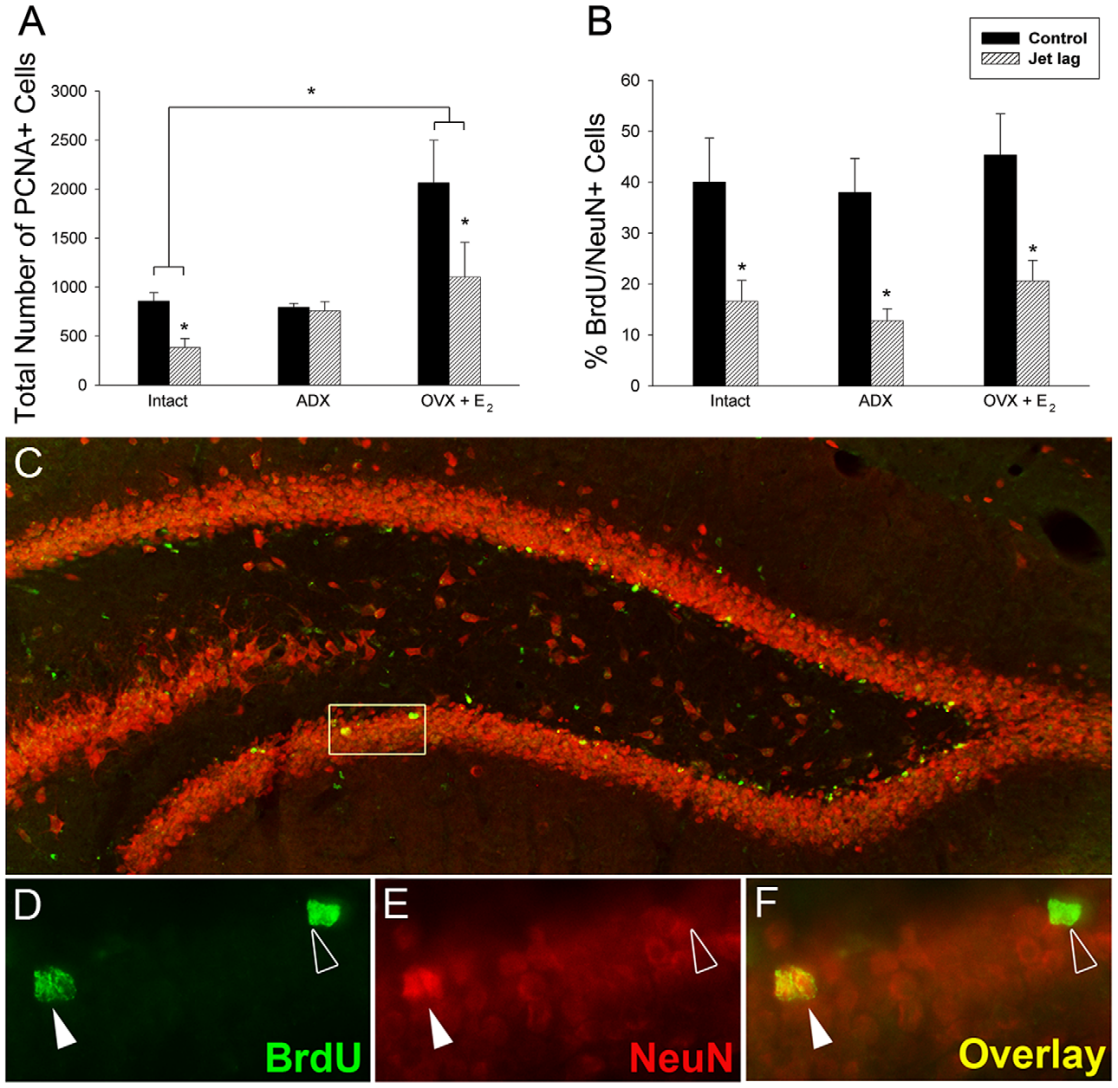
Jet lag adversely impacts PCNA immunostaining and neurogenesis in the dentate gyrus. (A) The number of PCNA-labeled cells in the granule cell layer was affected by the hormonal condition of the animal (*F_2,20_* = 4.014, *P = *0.03), with ovariectomy and estradiol replacement significantly increasing the number of labeled cells as compared to intact hamsters (*P = *0.04). Jet lag resulted in a significant decrease in the number of PCNA-labeled cells in both intact and OVX + E_2_ hamsters (*P = *0.007 and *P = *0.05, respectively by planned comparisons) while the number of PCNA-labeled cells in adrenalectomized animals was not affected by chronic temporal disruption (*P = *0.80). (B) Neurogenesis was decreased by jet lag (*F_1,21_* = 20.147, *P<*0.001), but there was no significant effect of hormone condition (*F_1,21_* = 0.228, *P = *0.80) and no interaction (*F_2,21_* = 0.231, *P = *0.80). Chronic jet lag resulted in a decrease in neurogenesis by >50% in intact, ADX, and OVX + E_2_ hamsters (*P = *0.01, *P = *0.007, and *P = *0.05, respectively; * *P<*0.05, n = 4/5 animals/group). (C–F) Sections were processed for double-label BrdU (green) and NeuN (red), a marker for mature neurons, and quantified at 400×. (C) Photomicrograph of the dorsal and ventral blades of the dentate gyrus. Cells were considered double-labeled when BrdU (D) and NeuN (E) co-localized in the same focal plane (F; yellow).

In intact hamsters, jet lag markedly suppressed cell proliferation, reducing the number of cells by approximately 50% (*P = *0.007; **Figure 1A**). Because jet lag and shift work are associated with elevated cortisol [16], and disruptions of the reproductive axis in human populations [10], and these alterations may influence cell proliferation and neurogenesis [37], [60], [61], we compared the effects of jet lag in intact and adrenalectomized hamsters given low, basal corticosterone replacement [49], [62] and ovariectomized females administered proestrous levels of estradiol (OVX+E_2_). The effect of jet lag on cell proliferation was abolished in adrenalectomized hamsters treated with corticosterone (*P = *0.80), suggesting that jet lag suppressed cell proliferation through activation of the HPA axis. As expected, ovariectomy and estrogen replacement increased the number of PCNA-labeled cells (*P<*0.05) [61]. However, jet lag decreased cell proliferation by the same magnitude as observed in intact animals (*P<*0.05). Together, these findings reveal a pronounced effect of jet lag on hippocampal cell proliferation, likely mediated by the HPA axis.

**Figure 2:**
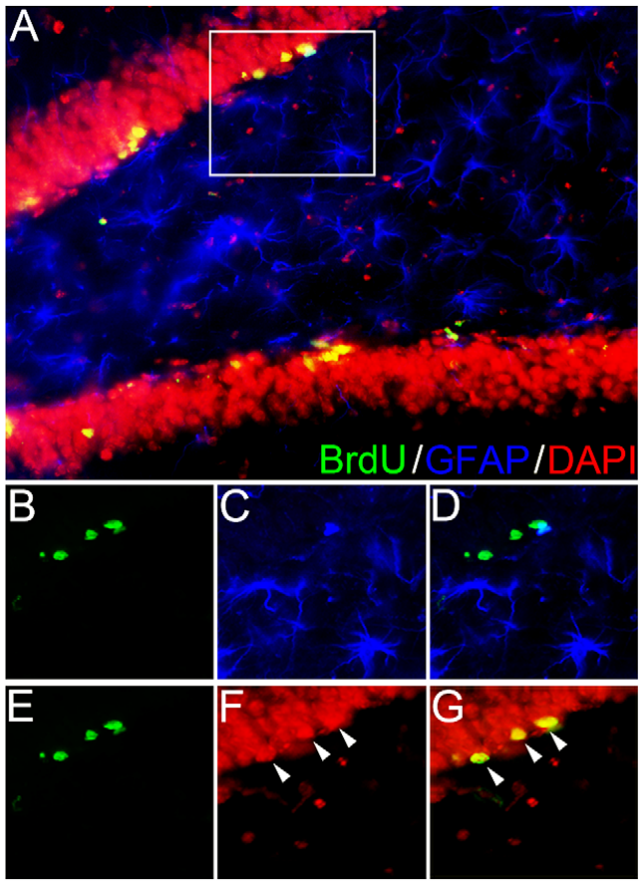
Gliogenesis is minimal in adult hippocampus. Hippocampal sections were immunostained for BrdU (green), GFAP (blue), and DAPI (red; color changed to red for purposes of visibility) to assess specificity of BrdU labeling and gliogenesis. (A) Representative photomicrograph (400×) of the dentate gyrus of the hippocampus expressing all three labels. Tissue was double-labeled immunofluorescently with antibodies against BrdU (B) and GFAP (C) to determine whether BrdU-labeled cells were glia (D). Approximately 2% of all BrdU-positive cells co-labeled with GFAP. In the triple-labeled image (D), three cells are labeled for BrdU, but do not co-express GFAP. Tissue was also labeled with fluorescent antibodies against BrdU (E) and DAPI (F) to ensure the specificity of BrdU labeling in mature neurons (G).

The consequences of jet lag on hippocampal cell survival and maturation were examined by quantifying BrdU expression in combination with NeuN to assess neurogenesis (**Figure 1B–F**) and GFAP to assess gliogenesis (**Figure 2** **and** **Table 1**). In all three conditions, jet lag reduced neurogenesis by >50% (**Figure 1B–F**). The magnitude of the suppression was not affected by adrenalectomy/ovariectomy and hormone replacement (*P*>0.05 in both cases). The same pattern of results was detected in the total number of BrdU-labeled cells (NeuN-positive *and* NeuN-negative) (*F_1, 21_* = 18.094, *P*<0.001; data not shown). Jet lag-induced suppression in neurogenesis did not impact the total volume of the granule cell layer (**Table 1**; *F_1, 21_* = 0.0126, *P* = 0.91). Thus, as with cell proliferation, jet lag negatively impacted neurogenesis, although this effect *was not* mediated by glucocorticoids, indicating that the jet lag-induced decrease in neurogenesis is independent of the ‘stress’ associated with experimental jet lag. This finding is not surprising as stress can differentially affect cell proliferation and survival [63]. There was no effect of jet lag (*F_1, 21_ = *0.136, *P = *0.72) or hormone condition (*F_2, 21_ = *0.318, *P = *0.73) on gliogenesis and less than 3% of BrdU-labeled cells were glia (2.2±0.89%) (**Figure 2** **and** **Table 1**).

**Table 1:** Jet lag does not affect gliogenesis and hippocampal volume.

	Intact		ADX		OVX + E_2_	
	Control	Jet Lag	Control	Jet Lag	Control	Jet Lag
**%BrdU/GFAP+ Cells**	3.0±3.0	3.2±3.2	0.0±0.0	2.4±2.4	2.0±1.2	1.6±1.6
**Granule Cell Layer Volume (mm^3^)**	13.26±0.96	14.48±0.75	14.73±1.10	14.19±0.71	15.25±0.67	14.11±0.56

### Jet Lag Results in Long-Term Deficits in Hippocampal-Dependent Learning and Memory

**Figure 3:**
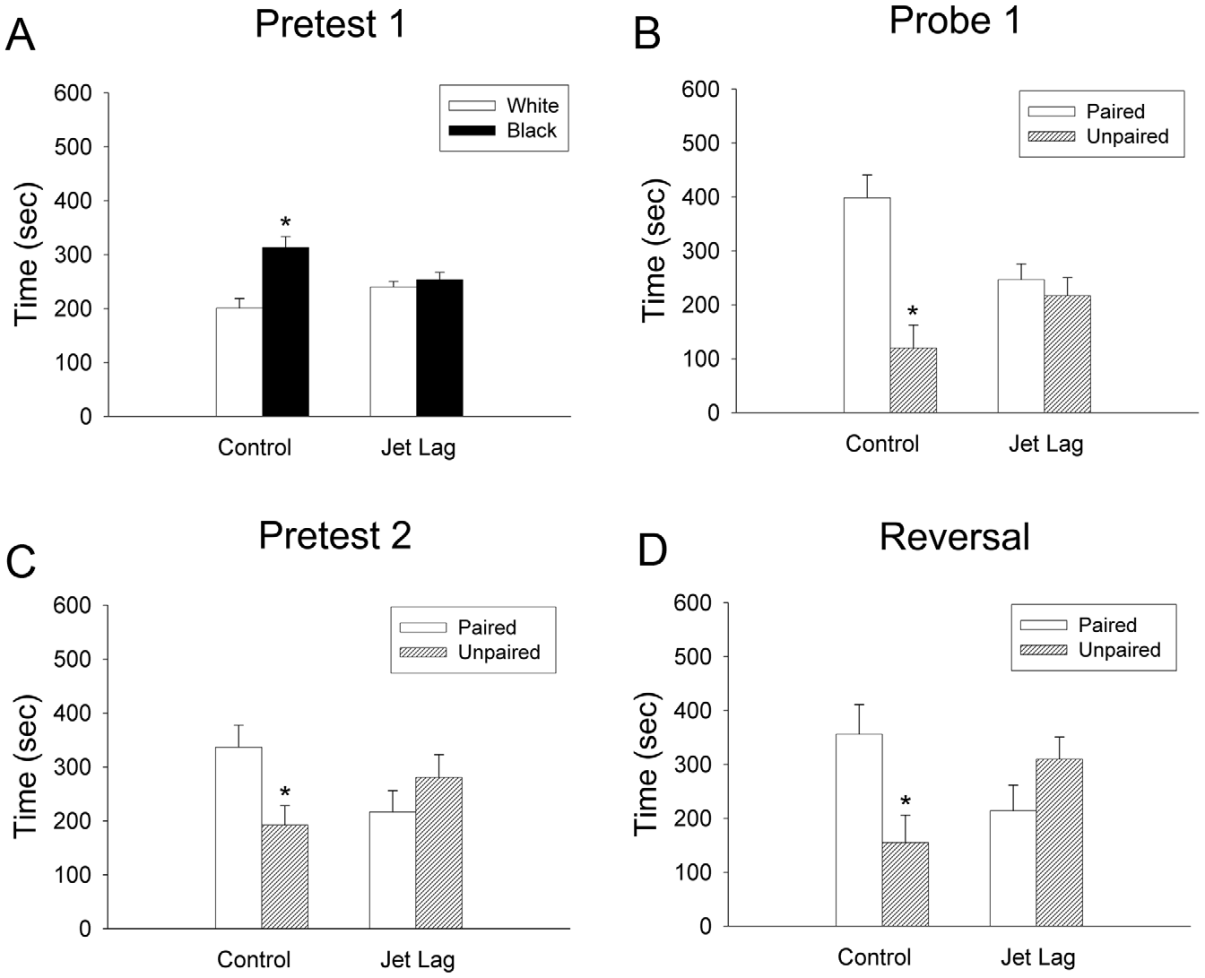
Jet lag disrupts hippocampal-dependent learning and memory. Control (n = 10) and Jet Lag (n = 10) hamsters were introduced to the conditioned place preference (CPP) paradigm at the same time of day throughout the experiment to control for time of day effects on learning and memory [15]. (A) Control hamsters exhibited a significant bias for the black chamber during initial exposure to the apparatus (Pretest 1) (*t_18_* = 4.193, *P<*0.001) whereas jet-lagged hamsters displayed no preference (*t_18_ = *0.805, *P = *0.43). (B) After training, control animals exhibited a significant preference for the chamber previously paired with the rewarding stimulus (Probe 1; *t_16_ = *4.620, *P<*0.001), whereas animals undergoing jet lag during training did not learn the task and showed an equal preference for both chambers (*t_16_ = *0.673, *P = *0.51). Jet-lagged hamsters were then returned to a static LD cycle for 28 days in order to re-establish entrainment of biological rhythms with the LD cycle. (C) Several weeks after re-entrainment, control animals maintained a preference for the previously paired chamber (*t_18_ = *2.2664, *P = *0.02) whereas jet-lagged hamsters continued to show no preference (*t_18_ = *1.113, *P = *0.28). All animals were then trained with the wheel being placed in the chamber opposite to that used in the first behavioral test. (D) Even after recovering from chronic temporal disruption, jet-lagged animals did not learn the task (*t_14_ = *1.532, *P = *0.15), whereas control animals learned to prefer the new chamber (*t_12_ = *2.692, *P = *0.02). * *P<*0.05.

Because reductions in neurogenesis are associated with deficits in learning and memory [29], [32], [64], we assessed the potential impact of jet lag-induced changes in neurogenesis on hippocampal-dependent memory. Using a conditioned place preference (CPP) paradigm [15], [55] in intact animals, the first learning and memory test commenced during the final 10 days of jet lag. The total duration of time the animals were actively exploring the apparatus during Pretest 1 did not differ between control (527.3±21.48 sec) and jet-lagged hamsters (541.8±18.71 sec)(*t_18_* = 0.509; *P* = 0.617), suggesting that any differences observed are not due to differences in alertness or motivation to explore. As expected, when initially exposed to the apparatus, control animals preferred the black compartment (Pretest 1; *t_18_* = 4.193, *P<*0.001), whereas jet-lagged animals exhibited no preference (*t_18_ = *0.805, *P = *0.43)(**Figure 3A**). If any animal exhibited a significant preference for one compartment during the Pretest, the running wheel was placed into the non-preferred compartment during the training sessions. To assess learning and memory, a probe trial was conducted in which the wheel was removed from the apparatus and the total time hamsters explored each compartment was recorded. The control animals developed a clear preference for the chamber previously containing the wheel, dwelling approximately 3 times longer in this chamber (Probe 1; *t_16_ = *4.620, *P<*0.001; **Figure 3B**). Despite identical training, jet-lagged animals were unable to perform the task, spending equal amounts of time in both chambers during the Probe trial (*t_16_ = *0.673, *P = *0.51). To examine the possibility that variable incongruence between CT and ZT in jet-lagged animals contributed to the deficits observed, we conducted a Levene test to determine if the variance differed between jet-lagged and control animals. Had the disparity between ZT (time of testing) and CT (time of activity) contributed to the deficits observed, then the variance should be greater in the jet-lagged group. The Levene test confirms that the variance did not differ between control and jet lag animals during Probe 1 (*P* = 0.903), suggesting that the disparity between ZT and CT did not contribute to the deficits uncovered.

It was anticipated that performance would be impaired in the midst of chronic temporal disruption, when rhythms in internal physiology and brain function are incongruous with external time. Given the suppressive actions of jet lag on hippocampal neurogenesis, we assessed whether the negative consequences of repeated circadian disruptions persist long after hamsters have re-synchronized their circadian rhythms to environmental time. Because hamsters recover from a 6-hr phase advance in approximately one week [1], [65], we retested the same hamsters used in the first CPP test 4 weeks after the cessation of the jet lag treatment to ensure that the animals were re-synchronized to the fixed LD cycle as activity monitoring was not logistically possible during this phase of testing. In Pretest 2, animals were placed into the empty apparatus to determine if they exhibited a preference for either chamber. The retention of the previous training would be reflected if hamsters exhibited a preference for the compartment that previously contained the activity wheel. Control hamsters showed a significant preference for the previously paired chamber (*t_18_ = *2.2664, *P = *0.02), whereas those that had been jet-lagged continued to show no preference (*t_18_ = *1.113, *P = *0.28; **Figure 3C**). This was expected, as the jet-lagged animals did not acquire the task during the first CPP session (**Figure 3B**). For the Reversal Experiment, the wheel was placed into the chamber opposite to that initially trained for each hamster during the first CPP test. By placing the wheel in the chamber opposite to the initial training, animals were required to acquire a new preference. Because control hamsters had acquired a preference in the initial CPP test, learning the association between the new chamber and wheel required that the animals override the previous memory. In contrast, because the jet-lagged hamsters did not acquire the initial preference, they might more readily acquire the new preference [66]. After training, control animals spent the majority of their time in the newly-trained chamber (*t_12_ = *2.692, *P = *0.02), but the previously jet-lagged hamsters did not develop a preference for either chamber (*t_14_ = *1.532, *P = *0.15; **Figure 3D**). This finding suggests that repeated phase advances negatively impact learning and memory well past the point of readjustment to the current time photoperiod.

### Repeated Jet Lag Transiently Activates the Stress Axis

**Figure 4:**
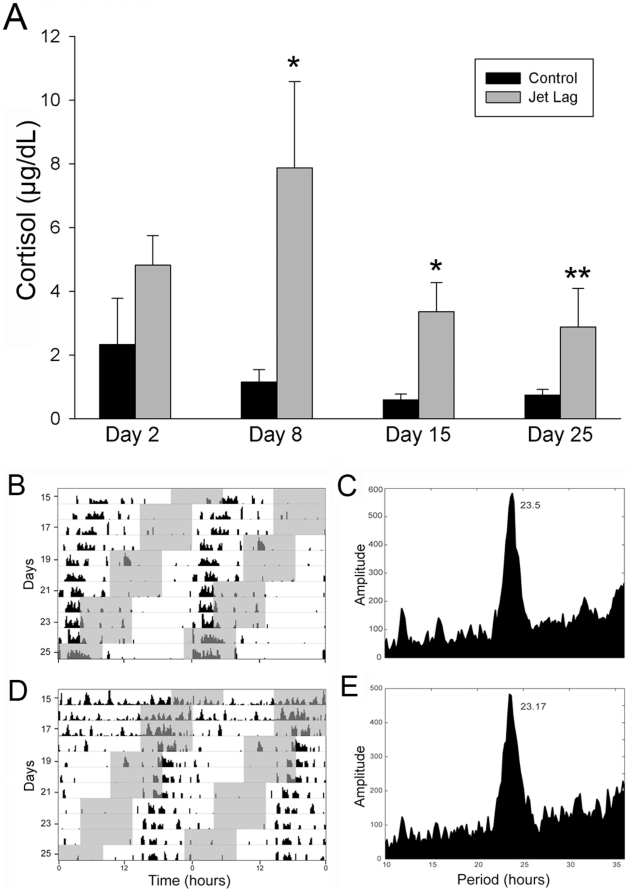
Jet lag transiently activates the HPA axis. (A) Jet-lagged animals (n = 7) exhibited increased concentrations of cortisol on days 8 and 15 (*P*<0.001 and *P* = 0.03) of the 25-day phase advance paradigm but not on the day following the first phase advance (*P* = 0.13) or the final day of the treatment (*P* = 0.11). The stress response in jet lag animals was attenuated throughout the course of the treatment with significantly lower concentration on day 25 compared to day 8 (*P* = 0.03). * Significantly greater than control animals (n = 7) at each time point, *P<*0.05. ** Significantly less than jet-lagged animals on Day 8 of sampling, *P<*0.05. (B–E) Jet-lagged animals ignore environmental light cues while maintaining rhythmic behavior. (B, D) Double-plotted actograms and (C, E) Fourier analysis of period length/rhythm amplitude of jet-lagged animals exposed to a chronic phase-advanced LD schedule. Records indicate that jet-lagged animals remain rhythmic (B, D), running with a period ∼24 hrs (C, E; *P*<0.01). Grey bars depict the dark phase of the LD cycle on these double-plotted activity records. Had animals been entrained to the LD cycle, activity would have been confined to these dark periods.

The impact of jet lag on *cell proliferation* was abolished by adrenalectomy and basal corticosterone replacement, suggesting that activation of the HPA axis may contribute to the impact of jet lag on this measure. To examine this possibility, a separate group of hamsters were exposed to the jet lag paradigm (n = 7) or to a fixed LD cycle (n = 7) and blood samples were collected throughout the treatment. There were no significant differences in glucocorticoid concentrations between control and jet-lagged animals on the day after the first 6-hr phase advance (Day 2; *P* = 0.13), with both groups exhibiting cortisol concentrations equivalent to non-stressed Syrian hamsters [67]. However, at subsequent time points, glucocorticoid concentrations of jet-lagged hamsters were higher than those of control animals (*F_1,36_* = 19.786, *P*<0.001; **Figure 4A**), with highest concentrations on Day 8 [67]. The increase in cortisol concentrations was attenuated during the second half of the phase advance treatment, with cortisol concentrations at the final time point significantly reduced from initial measurements (*P* = 0.03; **Figure 4A**), indicating that hamsters habituate to the repeated temporal adjustments. The duration of light exposure prior to sampling was not correlated with cortisol concentrations (R^2^ = 0.00000185, *P* = 0.995), suggesting that light exposure did not impact the cortisol measures differentially in jet-lagged and control animals.

### Jet Lag Leads to a Desynchrony between Internal and External Time

Because a functional circadian system is critical for normal hippocampal memory [68], we monitored the state of the circadian system throughout the jet lag treatment using an infrared monitoring system. In contrast to studies using *acute* phase adjustments, where animals re-synchronize their rhythms to the adjusted LD cycle, jet-lagged animals ‘ignored’ phase alterations in environmental time and exhibited non-entrained, circadian (∼24-hr) rhythms (**Figure 4B–E**). This finding suggests that deficits observed following repeated temporal disruptions result from desynchrony between internal and external time, rather than the absence of circadian organization. Had the jet lag treatment disrupted circadian functioning, locomotor behavior would have either become arrhythmic, exhibited a change in rhythm amplitude, or shown a rhythm period outside the normal circadian range.

Jet-lagged animals exhibited equivalent durations of activity (*alpha*) throughout the phase-advance treatment compared to activity while housed in a fixed LD cycle (**Table 2**; F_3,18_ = 1.559, *P* = 0.234). During fixed LD cycle and days 21–23 of the jet lag paradigm, the majority of activity was confined to the dark phase. In contrast, jet-lagged animals were significantly less active during the dark phase following the first phase-advancement (days 2–4; *P*<0.001) and mid-way through the jet lag treatment (days 15–17; *P*<0.001) compared to the fixed LD schedule (**Table 2**). This latter finding further indicates that jet-lagged animals did not entrain their activity to the LD cycle.

**Table 2:** Circadian analysis of hamsters maintained in a static light∶dark (LD) cycle and during days 2–4, 15–17, and 21–23 of the jet lag paradigm.

	Fixed LD	Days 2–4	Days 15–17	Days 21–23
***Alpha (hrs)***	6.881±0.678	5.150±1.369	6.384±0.876	7.103±0.657
**Nocturnality index**	0.647±0.038	0.138±0.027*	0.169±0.058*	0.542±0.075

* = significantly different from Fixed LD and Days 21–23 groups, *P*<0.05.

## Discussion

The present findings show, for the first time, that circadian disruptions lead to marked suppression of hippocampal cell proliferation and neurogenesis, associated with notable deficits in learning and memory. Adrenalectomy abolished the effects of jet lag on cell proliferation, suggesting that circadian disruptions impact this measure, in part, via HPA axis activation. In contrast, the pronounced suppression of neurogenesis is independent of jet lag-induced alterations in circulating glucocorticoid and sex steroid concentrations. Jet-lagged animals exhibited ∼24-hr rhythms, not synchronized with external time, suggesting that the observed deficits result from a desynchrony between internal physiology and external time, not from gross disruptions in internal rhythmicity. Additionally, the duration and amplitude of the activity/rest cycle was not impacted by the treatment, suggesting that the results are not a consequence of sleep deprivation as has been shown previously [69], [70]. Together, these results underscore the importance of circadian entrainment in maintaining optimal neural and cognitive functioning.

As indicated previously, the circadian system is an organized hierarchy with a master circadian pacemaker, the SCN, coordinating the timing of thousands of subordinate oscillators in the central nervous system (CNS) and periphery [2], [20]. Neural precursor cells (NPCs) from the dentate gyrus express circadian clock genes and disruption of the cellular clock results in abnormal NPC division and maturation [40]. Whereas most studies supporting a link between the circadian system and regulation of the cell cycle involve genetically disrupting the circadian clock, acute global temporal disruptions (e.g., jet lag) dysregulate the core clock mechanism without permanently altering molecular pathways [1]. As a result, we elected to use this model to explore the impact of circadian disruption without genetically altering the core molecular circadian clockwork. Whereas the present studies used phase advances to determine whether or not disrupting circadian organization impacted hippocampal physiology and function because this behavioral manipulation results in maximal circadian desynchrony, future studies should take into account alternating phase advances and phase delays in circadian rhythms to more accurately mimic the shift work and jet lag schedules experienced by human populations. Importantly, the results indicate that cell proliferation and neurogenesis can be suppressed by these temporal changes without genetic modifications of the circadian system, indicating that this phenomenon is worthy of further exploration.

In the present experiments, cortisol concentrations were elevated in jet-lagged hamsters throughout the course of the treatment, with recovery seen near the conclusion of the phase shifting (**Figure 4A**). On Day 8 of the jet lag paradigm, jet lag hamsters exhibited cortisol concentrations comparable to stress-induced values in this species [67]. On subsequent days, cortisol concentrations in the jet-lagged hamsters were lower than those seen in stressed animals but greater than daily maximum values [48]. This finding is consistent with the association between jet lag and glucocorticoid concentrations seen in women [16], [17]. Because cortisol should be lowest during the rest phase and may be impacted by light exposure, we assessed whether the duration of light exposure prior to sampling correlated with cortisol measures. Control hamsters were consistently sampled 7 hrs after lights on while sampling of jet-lagged hamsters occurred at CT7 and was variable relative to the LD cycle. There was no relationship between the two variables, suggesting that the duration of light preceding blood sampling, and the variable light exposure relative to the active phase in jet-lagged hamsters, was unlikely to impact cortisol concentrations. Additionally, the fact that the phase relationship between sampling and light differed throughout the one-month examination in jet-lagged animals, yet the increase in cortisol was maintained relative to controls until Day 25, further suggests that light was unlikely to impact interpretation of these findings.

Whereas jet lag reduced cell proliferation in the dentate gyrus of intact animals, this effect was abolished when circulating glucocorticoids were controlled through adrenalectomy and glucocorticoid replacement, suggesting that the jet lag-induced reduction in cell proliferation is mediated via activation of the HPA axis (**Figure 1A**). Because estradiol increases cell proliferation [71], it was not surprising that estradiol treatment increased PCNA cell labeling (**Figure 1A**), but it is noteworthy that the magnitude of PCNA suppression by jet lag was maintained in these animals and identical to that observed in intact animals.

Although these findings suggest that jet lag-induced suppression of hippocampal cell *proliferation* is mediated, at least in part, by increased glucocorticoid concentrations, other variables may contribute to this phenomenon. Neurotrophic factors, including BDNF and NGF, have been implicated in cell proliferation and/or survival [52], [72], [73]. In one study of acute jet lag, a single, 8-hr phase shift increased BDNF levels in the hippocampus [74]. Likewise, intracerebroventricular injections of NGF phase shift activity rhythms of Syrian hamsters [75] and increase survival of new cells [73]. Whereas both acute treatments increased these neurotrophic factors, it is possible that more chronic circadian disruptions suppress their expression. The extent to which different jet lag treatments impact neurotrophic factors has yet to be explored.

Unlike the impact of glucocorticoids on cell proliferation, circadian disruptions reduced neurogenesis by >50% in all groups, regardless of adrenal/glucocorticoid and ovary/estradiol status, indicating that the effect of jet lag on cell maturation is independent of increased HPA axis activity or alterations in gonadal steroids **(** **Figure 1B**). Reductions in the maturation of new neurons may reflect a decrease in production of new progenitor cells that differentiate into neurons, or a decrease in cell survival. Our data suggests that decreased hippocampal neurogenesis resulting from jet lag is a consequence of decreased cell survival, as adrenalectomy abolishes the effects of jet lag on cell proliferation, whereas reductions in neurogenesis persist. These findings are consistent with the notion that circadian cellular timing can directly impact cell survival [76]. Indeed, cell cycle genes, including *Wee-1, c-myc*, and *Cyclin-D1*, are regulated in a circadian manner [77], further suggesting that the regular timing of these genes contributes to normal cell functioning. As a result, disruption of proper circadian function may lead to alterations of the cell cycle, including modifications to cell survival and fate.

Reductions in newly-generated hippocampal neurons are associated with impairments in hippocampal-dependent learning and memory tasks [32]. While it is difficult to provide a direct cause-effect relationship between neurogenesis and learning and memory, as mentioned previously, many studies point to an association between the production of new hippocampal neurons and hippocampal-dependent cognitive processes [29], [30], [78], [79], [80]. Despite the fact that the inhibition of neurogenesis following a learning task consistently results in learning deficits, these findings must be interpreted cautiously as it is possible that the procedures used may not be restricted only to those cells born following a learning task or to hippocampal cell populations. However, these findings, combined with the fact that newly born hippocampal cells markedly increase following a hippocampal-dependent learning task [25], [26], [27], while learning tasks that are hippocampus *independent* do not [25], [28], provides strong evidence for a functional link between these two measures.

In the current study, when tested during the jet lag treatment, jet-lagged hamsters did not learn the conditioned preference task that control hamsters readily acquired (**Figure 3B**). Importantly, when trained one month following placement into a fixed LD cycle, jet-lagged animals were still unable to perform the CPP task (**Figure 3C and D**), suggesting that the impact of jet lag on learning and memory persists well after endogenous processes are re-synchronized to external time. Previous research indicates that both ZT and CT do not affect acquisition of the CPP as long as training and probe trials occur at the same ZT or CT [15]. Whereas all animals were trained and tested at the same ZT, the fact that jet-lagged animals did not entrain to the LD cycle resulted in an incongruence between CT and ZT in jet-lagged hamsters. Because we could not control for both ZT and CT, all training and probe trials were conducted at the same time of the light∶dark cycle (ZT). This procedure resulted in jet-lagged animals being trained during periods of their activity/rest cycle that varied relative to controls as well as training/testing trials occurring at non-24 hour intervals, potentially contributing to the deficits observed in the former group during the jet lag treatment [46]. Several points argue against this possibility. First, control and jet-lagged animals spent equal amounts of time actively exploring the apparatus during Pretest 1, indicating that all animals were equally motivated. Furthermore, had circadian phase impacted learning in the jet lag group, the variance in dwell time across animals during Probe 1 should be greater in jet lag compared with control animals, and this was not the case. Finally, jet-lagged animals remained unable to acquire the learning task one month after cessation of the jet lag when ZT and CT should be consistent between jet lag and control conditions.

It is possible that the cognitive impairment seen during phase advancements may result from increased cortisol production in jet-lagged animals (**Figure 4A**) [36]. The fact that the same deficits in learning and memory persist one month following maintenance in a static LD cycle, argues against this possibility. Whether or not reductions in neurogenesis persist one month after recovery from repeated phase shifts, suggesting a contribution to these continued deficits, represents an important question for future investigation. Notably, previous work has shown that repeated phase shifting *following* the acquisition of a passive avoidance task impairs retention, suggesting that circadian disruption can also retrogressively impair memory consolidation [14]. We now show that phase shifts at least one month *prior* to learning can also impair learning and memory. In agreement with these findings, one recent study found that more mild circadian manipulations, acute phase shifts either before or after contextual fear conditioning, attenuate recall of fear conditioned behavior without inducing sleep deprivation [46]. Together, these findings reveal that repeated temporal insults grossly impact learning and memory and suggest that resulting changes in hippocampal structure may have long-lasting consequences on cognitive function.

While the mammalian circadian clock can adjust to acute phase shifts in the light∶dark cycle, this adjustment requires several cycles to re-establish the relationship between the environment and the internal clock [44]. Thus, repeated phase advances, such as those seen in experimental jet lag, may result in more pronounced deficits in the ability to re-establish the appropriate phase relationship between the environment and internal physiology. The phase adjustments used in the present experiments result in circadian (∼24-hr) rhythms of activity that are not coordinated with external time (**Figure 4B–E** **;** **Table 2**). Throughout the jet lag treatment, animals exhibited equivalent bouts of activity but did not confine the majority of their activity to the dark phase of the LD cycle (**Table 2** **)**. In control animals, nocturnality index and alpha are highly correlated, with the majority of the activity bout being confined to the dark phase of the LD cycle. Because the phase-shifted animals were not synchronized to the light cycle, the duration of the activity/rest cycle was equal to control animals, but the percentage of activity confined to the dark phase was decreased. The former observation suggests that the learning and memory impairments observed in jet-lagged animals did not result from perturbations in circadian rhythmicity, but from desynchrony between internal physiology and external time. Although rhythmic locomotor behavior is a reliable indicator of SCN functioning, it is possible that extra-SCN oscillators (e.g., those in hippocampal cells) behave differently in response to jet lag than those in the master clock [20]. Although this possibility is unlikely, given the important role of clock genes in cell cycle regulation [77], this alternative hypothesis is worthy of exploration. It is noteworthy that sleep deprivation has also been shown to disrupt neurogenesis in both a glucocorticoid-dependent and independent manner [69], [70], [81]. In the present studies, it is unlikely that the effects of jet lag on hippocampal structure and function are mediated by disruptions in the activity/rest cycle, as the total duration of the active and inactive phases of the circadian cycle were not different between the fixed LD cycle and jet lag treatment (**Table 2**). However, future studies in which sleep architecture is monitored throughout the jet lag period are necessary to determine whether alterations in sleep contribute to the learning and memory deficits observed.

Together, our findings indicate that experimental jet lag has a pronounced, negative impact on cell proliferation and survival associated with significant deficits in hippocampal–dependent learning and memory. Importantly, the alterations in the circadian cycle were relatively minor in the present work compared to studies eliminating circadian function through lesions or genetic manipulations. These findings underscore the importance of considering the health consequences for individuals throughout the world engaging in rotating shift work or flexible schedules (e.g., medical residents, airline pilots, security personnel), maintaining poor sleep hygiene, or flying repeatedly across time zones, as the impact of these temporal insults may last well beyond the chronobiological challenges.

## Supporting Information

Figure S1 **BrdU Experimental Design.** Two weeks after recovery from adrenalectomy or ovariectomy, female hamsters were maintained in a fixed 14∶10 LD cycle (Control) or an LD cycle that was phase advanced by 6 hrs (Jet Lag), every three days, for 25 days. All animals were injected with BrdU the day following every other jet lag (JL) and were perfused 24 hrs after the last injection. The first three injections were implemented early in the experiment to assess neurogenesis (i.e., enough time for cells to mature into neurons) whereas injections were continued past this point to assess total cell proliferation during the experiment.
(TIF)

Figure S2 **Hypothetical Procedural Time Course.** Hypothetical activity records and procedural timelines for a control animal exhibiting ∼24‐hr rhythms in activity (black bars) that were confined to the dark phase (grey bars) of the light∶dark cycle and a jet‐lagged hamster exhibiting <24‐hr rhythm in behavior. For both control and jet lag animals, all CPP pretest, training and probe trials occurred 4 hrs prior to lights off (yellow bars; ZT10‐14;T1= training day 1). BrdU injections (red X) occurred the day after every other phase advance at ZT7 for all animals, with perfusions (green X) occurring 24 hrs after the final injection. Cortisol samples were acquired on Days 2, 8, 15, and 25 of the jet lag paradigm (blue arrow). For all animals, blood samples were collected at CT7 based on the individual animal's activity profile.
(TIF)
