# Supplementary figures and images for: Experimental ‘Jet Lag’ Inhibits Adult Neurogenesis and Produces Long-Term Cognitive Deficits in Female Hamsters
Source: PLoS One. 2010 Dec 1;5(12):e15267. doi: 10.1371/journal.pone.0015267 (PMC2995744; doi:10.1371/journal.pone.0015267)

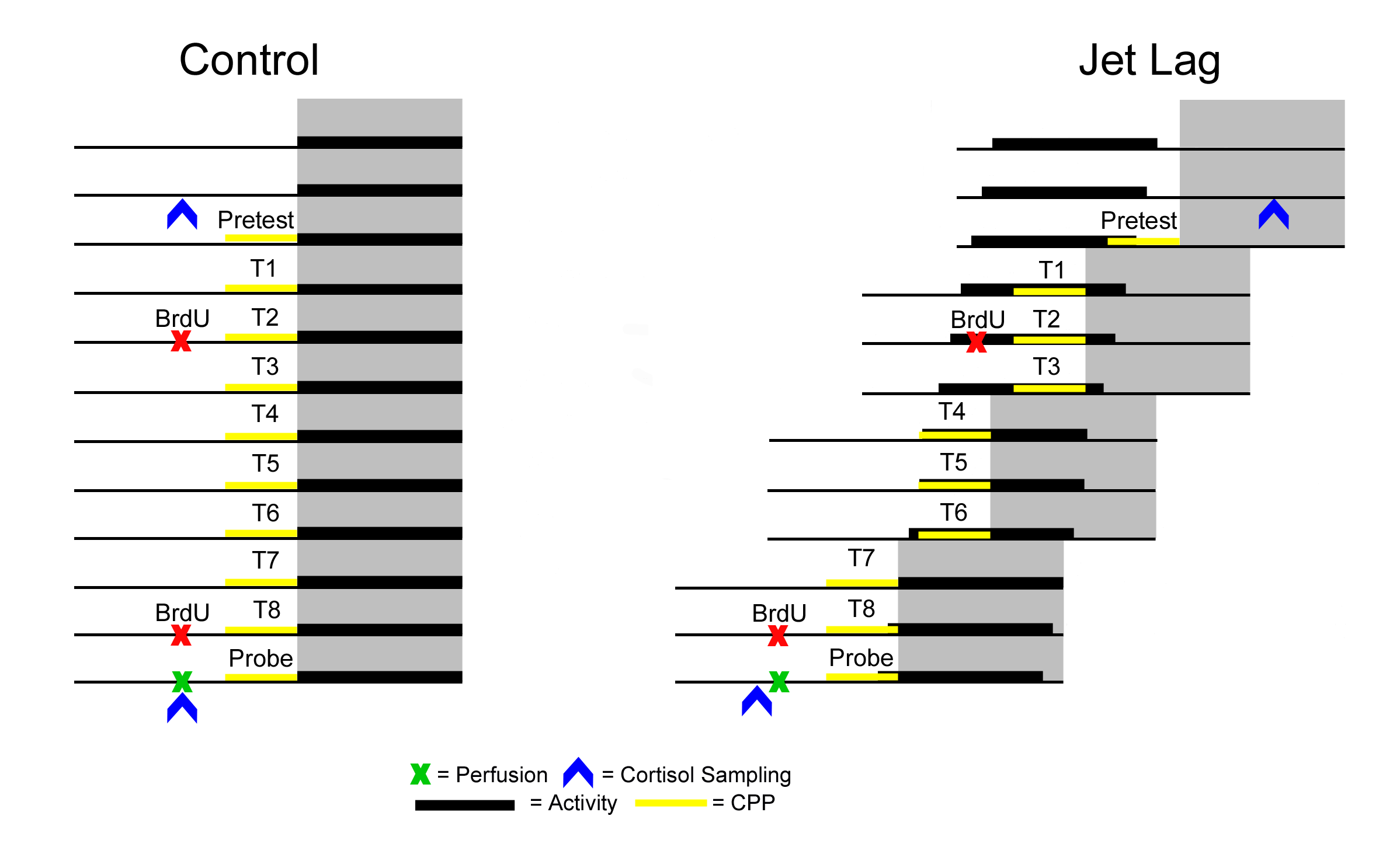

Supplement: Figure S2 — Hypothetical Procedural Time Course. Hypothetical activity records and procedural timelines for a control animal exhibiting ∼24‐hr rhythms in activity (black bars) that were confined to the dark phase (grey bars) of the light∶dark cycle and a jet‐lagged hamster exhibiting <24‐hr rhythm in behavior. For both control and jet lag animals, all CPP pretest, training and probe trials occurred 4 hrs prior to lights off (yellow bars; ZT10‐14;T1= training day 1). BrdU injections (red X) occurred the day after every other phase advance at ZT7 for all animals, with perfusions (green X) occurring 24 hrs after the final injection. Cortisol samples were acquired on Days 2, 8, 15, and 25 of the jet lag paradigm (blue arrow). For all animals, blood samples were collected at CT7 based on the individual animal's activity profile. (TIF) [file pone.0015267.s002.tif]
